# A Common Profile of Disordered Angiogenic Factor Production and the Exacerbation of Inflammation in Early Preeclampsia, Late Preeclampsia, and Intrauterine Growth Restriction

**DOI:** 10.1371/journal.pone.0165060

**Published:** 2016-10-19

**Authors:** Sebastian Kwiatkowski, Barbara Dołęgowska, Ewa Kwiatkowska, Rafał Rzepka, Andrzej Torbè, Magdalena Bednarek-Jędrzejek

**Affiliations:** 1 Department of Gynecology and Obstetrics, Pomeranian Medical University, Szczecin, Poland; 2 Department of Nephrology Transplantology and Internal Medicine, Pomeranian Medical University, Szczecin, Poland; 3 Department of Microbiology and Immunology, Pomeranian Medical University, Szczecin, Poland; Universite du Quebec a Trois-Rivieres, CANADA

## Abstract

Preeclampsia and intrauterine growth restriction are two separate disease entities that, according to numerous reports, share the same pathogenesis. In both, angiogenesis disorders and generalized inflammation are the dominant symptoms. In this study, we hypothesized that both diseases demonstrate the same profile in early preeclampsia, late preeclampsia, and intrauterine growth restriction patients, with the only difference being the degree of exacerbation of lesions. One hundred sixty-seven patients were enrolled in the study and divided into four groups: early preeclampsia, late preeclampsia, and intrauterine growth restriction groups, and one control group. Concentrations of the angiogenesis and inflammatory markers soluble fms-like tyrosine kinase receptor 1, placental growth factor, high-sensitivity C-reactive protein, and interleukin-6 were determined, and the behavior of these markers and correlations among them were studied. Higher concentrations of soluble fms-like tyrosine kinase receptor 1, high-sensitivity C-reactive protein, and interleukin-6 and a lower concentration of placental growth factor were observed in the study groups compared with the control group. No differences in concentrations of the studied markers were found among the study groups but significant correlations were observed. The higher values for the angiogenesis and inflammatory markers both in preeclampsia patients and patients with intrauterine growth restriction of placental origin compared with the control group suggest the existence of the same underlying disorders in the development of these pathologies. The observed mutual correlations for disordered angiogenesis and inflammatory markers are suggestive of a mutual relationship between these processes in the development of pathologies evolving secondary to placental ischemia. The same lesion profile was observed for both preeclampsia and ‘placental’ intrauterine growth restriction patients, which could be used in developing common diagnostic criteria for pregnant patients.

## Introduction

Preeclampsia and intrauterine growth restriction (IUGR) are the main causes of perinatal morbidity and mortality. These diseases affect 6–10% of pregnant women in North America. Impaired trophoblast invasion, and therefore perfusion disorder in the uteroplacental compartment, is seen as the pathogenesis of these conditions. It appears that early preeclampsia, late preeclampsia, and some cases of IUGR share the same placental pathology but show different exacerbations and demonstrate different clinical pictures in the mother and fetus [1]. It is suggested that this group should perhaps be given one name, ‘ischemic placental syndrome’, and treated as a single pathology that presents different clinical pictures.

Studies published to date indicate that a disordered blood supply to the placenta affects angiogenic, anti-angiogenic, and inflammatory factors synthesized by the placenta, and it is these factors that are responsible for the symptoms observed in the mother.

Currently, there are relatively specific markers that allow for assessment of the above processes. Angiogenesis disorder markers include soluble fms-like tyrosine kinase receptor 1 (sFlt-1) and placental growth factor (PlGF), while inflammatory markers include the already quite widely available high-sensitivity C-reactive protein (hsCRP) and interleukin (IL)-6.

This study examined whether the pathologies related to impaired trophoblast invasion (early preeclampsia before 34 weeks of gestation, late preeclampsia after 34 weeks of gestation, and IUGR) show similar disorders, which may indicate that they share the same pathogenesis. The aim was to assess whether disordered angiogenesis markers sFlt-1, PlGF, and the sFlt-1/PlGF ratio and inflammatory markers hsCRP and IL-6 differed between the study group and the control group, and among study subgroups (early and late preeclampsia and IUGR). Additionally, we sought to determine whether the disordered angiogenesis markers sFlt-1, PlGF, and PlGF as well as the sFlt-1/PlGF ratio correlate with the inflammatory markers hsCRP and IL-6 in early or late preeclampsia patients and in IUGR patients.

## Materials and Methods

The study group was 167 pregnant patients hospitalized between 2011 and 2015 at the Clinical Department of Obstetrics and Gynecology, Pomeranian Medical University, Szczecin, Poland. Patients: 42 patients diagnosed with early preeclampsia before 34 weeks of gestation (early); 41 patients diagnosed with late preeclampsia after 34 weeks of gestation (late); 41 patients suffering from IUGR; a control group of 43 patients with physiologically normal pregnancies (control). Each of the 4 groups of specific qualification criteria was subsequently expanded by further qualifying patients until each group reached the planned number of more than 40 patients.

Preeclampsia was diagnosed in patients with high blood pressure (24-h respiratory rate records) and new-onset proteinuria (>0.3 g/24-h in 24-h urine collection). In the absence of proteinuria, preeclampsia was diagnosed as hypertension in association with thrombocytopenia (platelet count <100 000/μL), impaired liver function (raised blood levels of liver aminotransferases to twice the normal concentration), new development of renal insufficiency (elevated serum creatinine >1.1 mg/dL), pulmonary edema, or new-onset cerebral or visual disturbances. IUGR was diagnosed as estimated fetal weight <10th percentile together with pulsatility index in the umbilical artery >95th percentile or cerebroplacental index <5th percentile. Patients diagnosed with a simultaneous IUGR were excluded. In the IUGR group, no presence of higher arterial blood pressure and/or proteinuria was observed. Cases of IUGR caused by a genetic factor, infection (diagnostic amniocentesis was performed on some patients, while others were analyzed after delivery), or maternal factors were excluded from the IUGR group.

Inclusion criteria for the study were: (a) early preeclampsia, patients admitted to the Clinical Department between 24 and 33 weeks for a singleton pregnancy with symptoms of preeclampsia, without co-morbidities; (b) late preeclampsia, patients admitted to the Clinical Department between 34 and 40 weeks for a singleton pregnancy with symptoms of preeclampsia, without co-morbidities; (c) IUGR, patients between 24 and 40 weeks of gestation with symptoms of IUGR but no symptoms of preeclampsia, after fetal and maternal causes were ruled out. Some patients were only included upon delivery, after fetal genetic causes and fetal infection were ruled out; and (d) control group, patients between 24 and 40 weeks of gestation without any accompanying pathology or chronic disease, hospitalized at our Clinical Department of Obstetrics and Gynecology.

Biochemical determinations were made using standardized kits that make use of the ELISA immunofluorescence technique. In each of the studied women, sFlt-1, PlGF, the sFlt-1/PlGF ratio, hsCRP, and IL-6 were determined. Blood for assessment of the studied parameters was drawn with the patient’s informed consent, and was centrifuged within 30 min of the sample being taken and stored at −80°C until analysis. Blood for routine biochemistry tests was sampled into standard test tubes and transported to the Central Hospital’s laboratory immediately. The results were subjected to statistical analysis using StatSoft software (Poland).

### Statistical analysis

Assessment of the normal distribution of continuous variables (Kolmogorov–Smirnov), which was the case for most variables, showed deviation from normal (log normal) schedule parameters. The routine biochemistry tests and the parameters characterizing the group (Table 1) were shown using group size, the median, and the minimum and maximum values. For comparison of the aforementioned parameters between the study group and the control group, a Mann-Whitney U-test was used. The study parameters were characterized by sample size, arithmetic mean, and standard deviation. Distributions of the aforementioned parameters were normalized by being converted into logarithmic continuous variables. To evaluate differences between studied parameters, Student’s *t-*tests were used for unpaired variables (Table 2) and analysis of variance (ANOVA) and multivariate analysis of variance were used for multivariable systems followed by multiple comparison *post-hoc* Tukey’s tests. The assumptions required for ANOVA (normality distribution and homoscedasticity) were not violated in any way that could have impaired the credibility of the F statistic. Strengths of correlations between parameters were measured using Pearson’s correlation coefficient (variables with normal distribution) or Spearman’s rank correlation coefficient (variables deviating from normal distribution).

**Table 1:** Characteristics and a statistical analysis of routine diagnostics of the study groups (Mann-Whitney U-test).

Group	Early (n-42)			Late (n-41)			IUGR (n-41)			Control (n-43)	
Parameters	Median	(min–max)	p-value	Median	(min–max)	p-value	Median	(min–max)	p-value	Median	(min–max)
Parity	1	(1–3)	ns	1	(1–5)	ns	1	(1–5)	ns	1	(1–4)
Gravidity	1	(1–6)	ns	1	(1–5)	ns	1	(1–5)	ns	1	(1–5)
Age (years)	32	(22–44)	ns	33	(19–42)	ns	31	(17–42)	ns	31	(18–41)
Gestation (weeks)	31	(26–33)	ns	37	(34–40)	<0.001	36	(22–41)	<0.001	32^1^	(24–40)
Height (cm)	165	(156–176)	ns	165	(156–174)	ns	165	(68–179)	ns	165	(150–181
Weight (kg)	75	(64–125)	ns	75	(60–129)	ns	69	(52–120)	ns	70	(53–129)
RR dia (mmHg)	110	(70–146)	<0.001	110	(84–138)	<0.001	75	(60–130)	ns	70	(50–90)
RR sys (mmHg)	182	(110–225)	<0.001	170	(140–228)	<0.001	133	(90–160)	<0.01	118	(120–150)
Proteinuria (g/24-h)	2.01	(0.4–16.23)	<0.001	1.13	(0.60–9.10)	<0.001	0.00	(0.00–0.00)	ns	0.00	(0.00–0.00)
Fetal weight (g)	1180	(590–2380)	<0.001	2850	1335–3940	<0.001	2130	(670–2700)	<0.001	3230^2^	(2700–3950)
PT (s)	10	(8.80–11.30)	ns	10.20	(8.6011.66)	<0.05	10.26	(9.30–13.30)	ns	10.30	(9.20–11.90)
D-dimers (ng/mL)	3760	(683–10000)	<0.001	2921.45	(582–10000)	<0.01	1116.70	(301.5–3198)	<0.001	892.50	(465.7–1887)
APTT (s)	27.2	(21.50–85.60)	<0.01	27.55	(20.40–32.30)	<0.01	26.75	(19.80–34.60)	<0.01	29.00	(24.70–33.90)
Fibrinogen (g/L)	3.65	(1.70–7.10)	ns	4.40	(2.50–10.60)	ns	4.50	(2.60–10)	ns	4.30	(2.10–5.90)
WBC (×10^9^/L)	11.87	(7.32–18.07)	<0.001	12.48	(7.33–18.00)	ns	11.16	(6.56–19.77)	ns	10.67	(6.17–18.87)
Ht (%)	0.34	(0.25–0.40)	<0.01	0.33	(0.25–0.42)	ns	0.35	(0.26–0.42)	ns	0.34	(0.27–0.40)
Hb (mmol/L)	7.14	(4.80–9.00)	ns	6.87	(5.10–9.00)	ns	7.20	(5.20–8.90)	ns	7.40	(5.80–8.50)
RBC (×10^12^/L)	3.88	(2.81–4.59)	ns	3.73	(2.62–4.50)	ns	3.95	(3.15–4.80)	ns	3.92	(3.26–4.57)
PLT (×10^9^/L)	139	(39–311)	<0.01	161.5	(40–367)	ns	203.0	(116–348)	ns	204.0	(107–351)
LDH (U/L)	365	(216–2595)	<0.001	251.0	(157–1136)	<0.001	197.5	(145–366)	ns	180.0	(100–550)
ALT (U/L)	40	(6–692)	<0.001	21.5	(6.0–704)	ns	16.0	(6–102)	ns	19.5	(7–73)
AST (U/L)	37	(11–1074)	<0.001	24.5	(14.0–900)	<0.001	20.0	(9–50)	ns	19.0	(10–37)
UA (μmol/L)	6.4	(2.90–10.70)	<0.001	6.55	(4.20–12.00)	<0.001	4.85	(2.60–8.50)	ns	4.30	(2–8.50)

P-value statistically significant differences between the study groups and the control group. Early, patient group with early-onset preeclampsia; Late, patient group with late-onset preeclampsia; IUGR, patient group with intrauterine growth restriction; RR dia, respiratory rate diastolic blood pressure; RR sys; respiratory rate systolic blood pressure; PT, prothrombin time; APTT, activated partial thromboplastin time; WBC, white blood cell count; Ht, hematocrit; Hb, hemoglobin; RBC, red blood cell count; PLT, platelet count; LDH, lactate dehydrogenase; ALT, alanine transaminase; AST, aspartate transaminase; UA, uric acid; ns, not significant;

^1,^ gestational age at inclusion;

^2,^ neonatal birth weight after delivery between the 38^th^ and 42^nd^ weeks of pregnancy.

**Table 2:** Analysis of study parameters between the study groups and the control group using the Student’s *t*-test.

Parameter	Control (*n* = 43)	Early (*n* = 42)	Late (*n* = 41)	IUGR (*n* = 41)
	Mean ± SD	Mean ± SD; p-value	Mean ± SD; p-value	Mean ± SD; p-value
log sFlt-1	3.23 ± 0.26	3.96 ± 0.27; p<0.0001	3.98 ± 0.30; p<0.0001	3.64 ± 0.38; p<0.0001
log PlGF	2.58 ± 0.32	1.31 ± 0.26; p<0.0001	1.77 ± 0.28; p<0.0001	1.93 ± 0.48; p<0.0001
log sFlt-1/PlGF	0.64 ± 0.45	2.65 ± 0.36; p<0.0001	2.20 ± 0.38; p<0.0001	1.71 ± 0.69; p<0.0001
log IL-6	0.63 ± 0.21	0.86 ± 0.23; p<0.0001	0.86 ± 0.33; p<0.001	0.73 ± 0.30; ns
log hsCRP	−0.33 ± 0.43	0.76 ± 0.23; p<0.0001	0.97 ± 0.20; p<0.0001	0.79 ± 0.40; p<0.0001

P-value statistically significant differences between the study groups and the control group. SD, standard deviation; Early, patients with early-onset preeclampsia; Late, patients with late-onset preeclampsia; IUGR, patients with intrauterine growth restriction; sFlt-1, fms-like tyrosine kinase receptor 1; PlGF, placenta growth factor; log sFlt-1, log sFlt-1 concentration in plasma; log PlGF, log PlGF concentration in plasma; log sFlt-1/PlGF; log sFlt-1/PlGF ratio; log IL-6, log interleukin 6 concentration in plasma; log hsCRP, log high-sensitivity C-reactive protein concentration in plasma.

### Ethical considerations

The study was performed with the approval of the Bioethics Committee at Pomeranian Medical University (No. n.KB-0012/122/12), and was conducted in accordance with the principles expressed in the Declaration of Helsinki. All participants provided written informed consent to participate in the study.

## Results

In the early and late preeclampsia groups hypertension, the value of proteinuria, and the activity of lactate dehydrogenase, aspartate transaminase and uric acid were significantly different to those in the control group (p<0.001). No such differences were identified in the IUGR group. Only in the early preeclampsia group were differences found compared to the control group in respect of the activity of alanine transaminase, platelet count, the hematocrit and WBC. In all the three study groups, D-dimers represented significantly higher values, while APTT was significantly shortened compared to the control group (p<0.01) (Mann-Whitney U-test, Table 1).

A statistical significant increase in the concentration of sFlt-1 was observed in the early preeclampsia group, the late preeclampsia group, and the group of patients with IUGR but without preeclampsia compared with the control group (p<0.0001). PlGF was significantly lower in all three study subgroups compared with the control group (p<0.0001). The sFlt-1/PlGF ratio was significantly higher in preeclampsia patients and IUGR patients compared with the control group (p<0.0001). Concentrations of inflammatory markers IL-6 and hsCRP were significantly higher in the study subgroups compared with the control group (Student’s *t*-test, Table 2).

**Fig 1:**
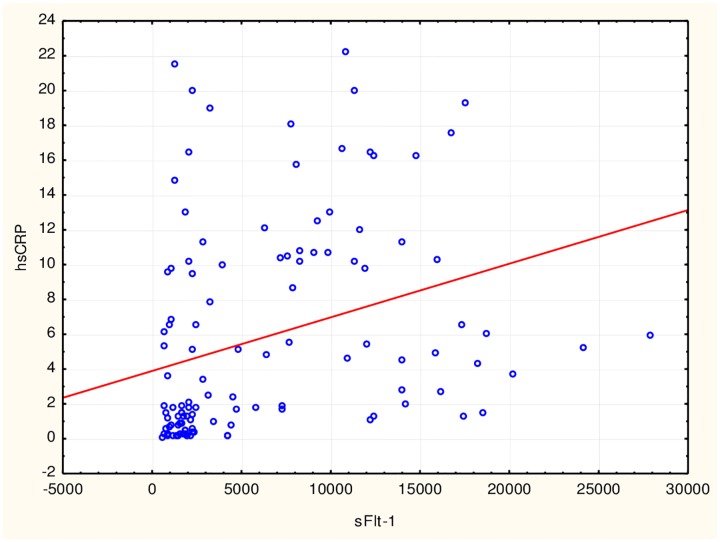
Correlation between high-sensitivity C-reactive protein (hsCRP) and fms-like tyrosine kinase receptor 1 (sFlt-1). A positive correlation between sFlt-1 and hsCRP was observed in the study population (r = 0.47, p<0,001) using Spearman’s correlation analysis.

Correlations were assessed for the entire study population. The correlation between concentrations of sFlt-1 and PlGF showed a statistically significant negative correlation (p<0.0001, r = −0.56). A positive correlation of similar power was observed between sFlt-1 and hsCRP concentrations (p<0.0001, r = 0.47) (Fig 1), and a negative correlation between concentrations of PlGF and hsCRP was observed (p<0.0001, r = −0.55). There was a statistically significant positive correlation between the sFlt-1/PlGF ratio and IL-6 (p<0.001, r = 0.33) and a negative correlation between PlGF and IL-6 (p<0.0001, r = −0.36). There was a significant positive correlation between hsCRP and IL-6 (p<0.01, r = 0.28).

**Fig 2:**
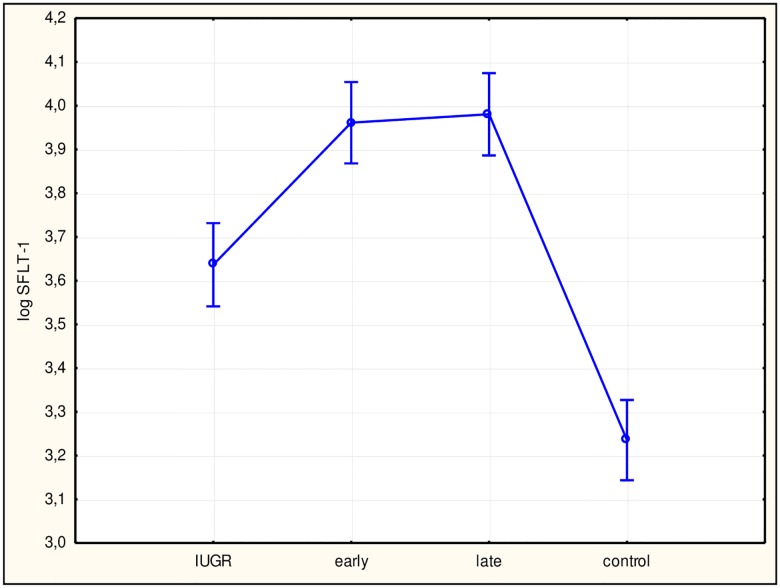
Analysis of variance of soluble fms-like tyrosine kinase receptor 1 (sFlt-1) in the study groups. The difference between sFlt-1 concentrations of the study groups with a 95% confidence interval was p<0.0001 and F = 55.624. Interactions between groups for sFlt-1 usingTukey’s test (examining statistically significant differences) were: intrauterine growth restriction (IUGR)/control (p<0.0001); early/control (p<0.0001); and late/control (p<0.0001). log sFlt-1, log sFlt-1 concentration in plasma; early, early-onset preeclampsia; late, late-onset preeclampsia.

**Fig 3:**
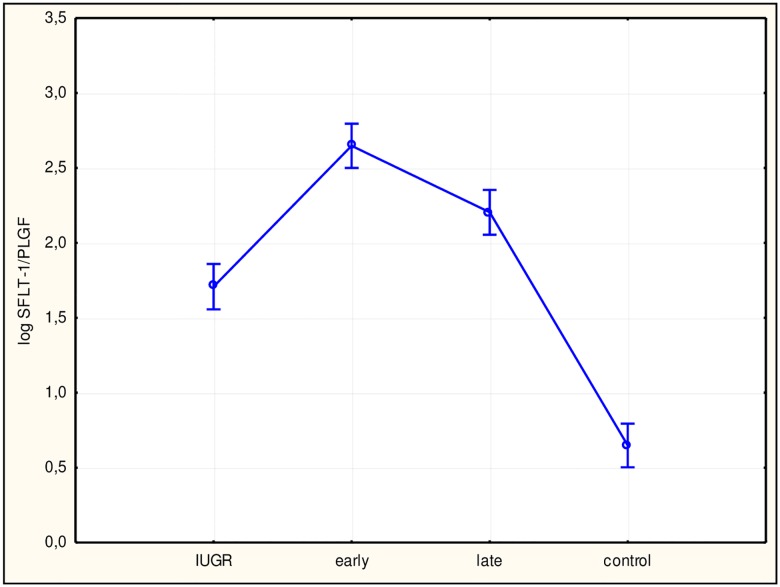
Analysis of variance for the soluble fms-like tyrosine kinase receptor 1 (sFlt-1) and placental growth factor (PlGF) ratio in the study groups. The difference between the sFlt-1/PlGF ratio in the study groups with a 95% confidence interval was p<0.0001, F = 133.57. Interactions between groups for the sFlt-1/PlGF ratio using Tukey’s test (examining statistically significant differences) were: intrauterine growth restriction (IUGR)/control (p<0.0001); early/control (p<0.0001); late/control (p<0.0001). log sFlt-1/PlGF, log sFlt-1/PlGF ratio; early, early-onset preeclampsia; late, late-onset preeclampsia.

**Fig 4:**
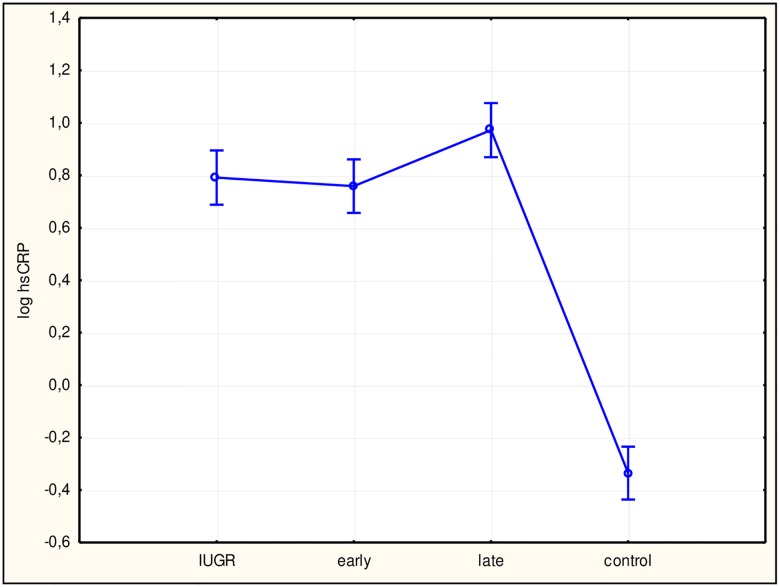
Analysis of variance for high-sensitivity C-reactive protein (hsCRP) in the study groups. The differences between hsCRP concentrations in the study groups with a 95% confidence interval was p<0.0001, F = 134.29. Interactions between groups for hsCRP using Tukey’s test (examining statistically significant differences) were: intrauterine growth restriction (IUGR)/control (p<0.0001); early/control (p<0.0001); late/control (p<0.0001). log hsCRP, log hsCRP concentration in plasma; early, early-onset preeclampsia; late, late-onset preeclampsia.

**Fig 5:**
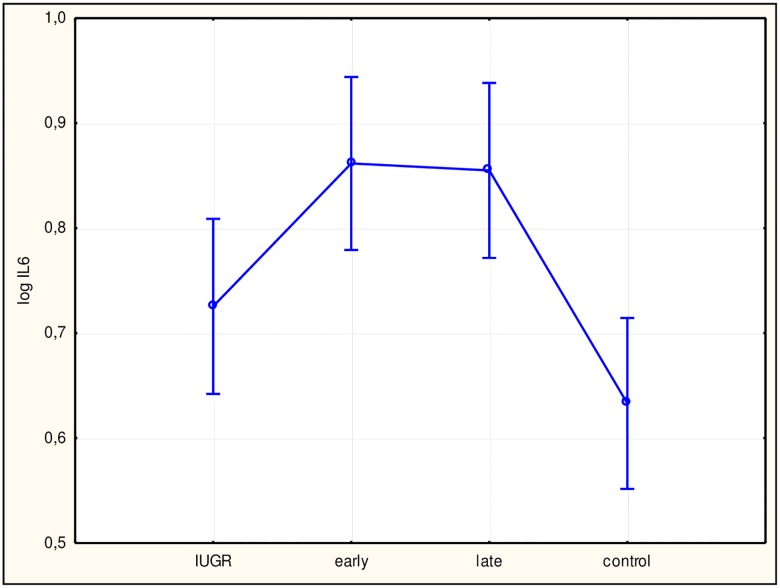
Analysis of variance for interleukin (IL)-6 in the study groups. The difference between IL-6 concentrations in the study groups with a 95% confidence interval was p<0.0001, F = 7.0022. Interactions between groups for IL-6 using Tukey’s test (examining statistically significant differences) were: intrauterine growth restriction (IUGR)/control (not significant); early/control (p<0.0001); late/control (p<0.001). log IL-6, log interleukin 6 concentration in plasma; early, early-onset preeclampsia; late, late-onset preeclampsia.

**Fig 6:**
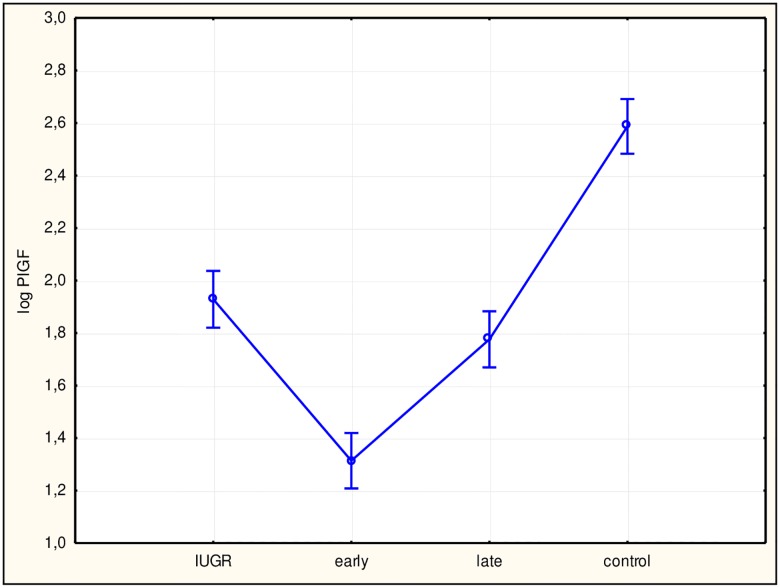
Analysis of variance for placental growth factor (PlGF) in the study groups. The difference between PlGF concentrations in the study groups with a 95% confidence interval was p<0.0001, F = 98.339. Interactions between groups for PlGF using Tukey’s test (examining statistically significant differences) were: intrauterine growth restriction (IUGR)/control (p<0.0001); early/control (p<0.0001); late/control (p<0.0001). log PlGF, log PlGF concentration in plasma; early, early-onset preeclampsia; late, late-onset preeclampsia.

To assess differences among study subgroups and between subgroups and the control group, ANOVA was performed, which returned statistically significant results for all factors. Subsequently, we performed Tukey’s test. Values for sFlt-1 (Fig 2), the sFlt-1/PlGF ratio (Fig 3), hsCRP (Fig 4), and IL-6 (Fig 5) in all study subgroups were higher than that of the control group (p<0.05). For PlGF (Fig 6), values for all subgroups were lower than that of the control group (p<0.05).

## Discussion

Preeclampsia and IUGR are the main causes of perinatal morbidity and mortality. In many cases, IUGR occurs together with preeclampsia. Frequently, IUGR occurs on its own, presenting clinical symptoms in the fetus, but the mechanism behind the development of the disease is the same as in preeclampsia [1]. In previous studies [2, 3], examined placentae from IUGR patients showed characteristics of abnormal uterine artery remodeling, and contained numerous lesions of the chorionic villi, suggesting reduced uteroplacental circulation. Lesions included syncytiotrophoblast knots, villous fibrosis, a reduced volume of villi, and non-specific inflammatory infiltration (villitis of unknown etiology). The lesions correlated with changes in uterine artery blood flow assessed using Doppler sonography [2–4].

A combination of abnormal uterine artery flow and the histopathological features of abnormal uterine artery remodeling are also typical of preeclampsia. It appears that the two diseases (IUGR and preeclampsia) share the same etiology. Recent studies have reported similarities in etiologies between preeclampsia and IUGR by examining smooth muscle cells from chorionic villi. In both diseases, reduced expression of cystathionine gamma-lyase, responsible for the synthesis of the vasodilator hydrogen sulfide, was shown to exist. The diminished expression of hydrogen sulfide provides increased resistance in placental vessels, which translates into abnormal profiles obtained with uterine artery Doppler sonography [5]. Similarly, villitis of unknown etiology has been identified in both diseases. Inflammatory infiltration mainly comprises histiocytes and lymphocytes [6, 7]. It is suggested that perhaps this group should be given a single name, ‘ischemic placental syndrome’, and treated as one pathology that presents different clinical symptoms. A shared pathogenesis of these diseases is also suggested by studies assessing other parameters showing increased in early and late preeclampsia and IUGR [8].

An abnormal placenta produces angiogenic and anti-angiogenic factors in a disordered manner. In diseases related to placental ischemia, reduced production of PlGF (a factor that stimulates angiogenesis) and increased production of its antagonist (sFlt-1) has been reported [9–12]. Apart from the factors affecting angiogenesis, an ischemic placenta also produces immunologic factors (tumor necrosis factor alpha, IL-6, and IL-8) which lead to generalized inflammation [13]. In the current study, we observed elevated concentrations of sFlt-1, hsCRP, and IL-6 in early preeclampsia, late preeclampsia, and IUGR groups compared with the control group. In the study subgroups, the concentration of PlGF was lower than that of the control group. Similar results have been reported previously. For example, Husse reported that preeclampsia and IUGR groups had higher angiogenesis markers than the control group [14]; Alahakoon reported a shared profile for angiogenesis markers in both preeclampsia and IUGR patients [15]; and the results of a study by Schoofs were the same [16].

Placental pathology involves secretion by the placenta of factors that exacerbate inflammation. CRP is an acute-phase protein [17] mainly synthesized and secreted by hepatocytes, but local synthesis and secretion occur in other cells, such as macrophages and smooth muscle cells [18, 19]. IL-6 is the main stimulator of the production of CRP. It is secreted by activated leukocytes and smooth muscle cells in response to infection or injury, but also by an abnormal ischemic placenta [13, 20, 21]. In the current study, we observed elevated concentrations of hsCRP in the preeclampsia and IUGR groups compared with the control group of healthy pregnant woman. Farzadnia *et al*. reported high hsCRP content in severe preeclampsia groups, in agreement with our results [22]. Saarelainen *et al*. reported that the hsCRP content was higher in patients suffering from gestational hypertension during pregnancy than in the same patients after pregnancy [23]. Can *et al*. also reported high hsCRP levels in preeclampsia patients [24].

From a review of the literature, we were unable to find any reports on hsCRP in IUGR patients. Only Cheng *et al*. used this marker and confirmed its value when forecasting the occurrence of IUGR already in the first trimester of gestation [25]. In those studies, IL-6 levels were higher in the preeclampsia and IUGR groups compared with the control group. The results align with findings from other studies of preeclampsia patients; for example, Udenze *et al*. [26] and Rahardjo *et al*. [27]. The results of a study of IUGR patients by Elfayomy *et al*. were similar to our own [28].

In ischemic placental syndrome patients, the exacerbation of inflammation correlates with the scale of production of anti-angiogenic and angiogenic factors, as evidenced by our findings of a positive correlation between the sFlt-1 concentration and the sFlt-1/PlGF ratio on the one hand and hsCRP and IL-6 concentrations on the other, as well as a negative correlation between the PlGF concentration on the one hand and hsCRP and IL-6 concentrations on the other. Additionally, IL-6 is the main driver behind the production of CRP, as suggested by the observed positive correlation between IL-6 and hsCRP. Reamer *et al*. reported a positive correlation between sFlt-1 and CRP in patients with HELLP syndrome (hemolysis, elevated liver enzymes, low platelet count), but found no correlation is women with preeclampsia [29]. Ouyang *et al*. observed the same relationship in preeclampsia patients.

We were not able to find any reports in the literature on research that examined the markers studied in the current study that included a group of IUGR patients [30]. In the current study, we observed an elevated concentration of the anti-angiogenic factor sFlt-1 and a decreased concentration of the angiogenic factor PlGF in the study group compared with the control group. Similarly, we observed increased levels of inflammatory factors IL-6 and hsCRP in the study group compared with the control group. The study group included three subgroups, these being early preeclampsia, late preeclampsia, and IUGR patients. Tukey’s test (preceded by ANOVA) was used to examine whether the subgroups differed from each other and from the control group. sFlt-1, hsCRP, and IL-6 in each subgroup were higher than that of the control group. We found no differences among the study subgroups. The PlGF level was lower in all subgroups compared with the control group, and there were no differences among subgroups. These findings suggest that the three disease types, which are still believed to be separate conditions, share the same etiology.

## Conclusions

The higher values observed for the angiogenesis and inflammatory markers both in preeclampsia patients and patients with IUGR of placental origin compared with the control group suggest the existence of the same underlying disorders in the development of these pathologies. The observed mutual correlations for disordered angiogenesis and inflammatory markers are suggestive of a mutual relationship between these processes in the development of the pathologies evolving secondary to placental ischemia. We found the same lesion profile for both preeclampsia and ‘placental’ IUGR patients, which could be used in developing common diagnostic criteria for pregnant patients.

## Supporting Information

S1 File Source data. Values of study parameters (sFlt-1, PlGF, IL-6, hsCRP) and weeks of pregnancy in the study groups.
(XLSX)
